# Genome-wide binding of the basic helix-loop-helix myogenic inhibitor musculin has substantial overlap with MyoD: implications for buffering activity

**DOI:** 10.1186/2044-5040-3-26

**Published:** 2013-11-01

**Authors:** Kyle L MacQuarrie, Zizhen Yao, Abraham P Fong, Stephen J Tapscott

**Affiliations:** 1 Human Biology Division, Fred Hutchinson Cancer Research Center, 1100 Fairview Ave N C3-168, Seattle WA 98109, USA; 2 Molecular and Cellular Biology Program, University of Washington, Seattle WA 98105, USA; 3 Clinical Research Division, Fred Hutchinson Cancer Research Center, Seattle WA 98109, USA; 4 Department of Pediatrics, University of Washington School of Medicine, Seattle WA 98105, USA; 5 Department of Neurology, University of Washington, Seattle WA 98105, USA

**Keywords:** Rhabdomyosarcoma, musculin, MyoD, myogenic inhibitor

## Abstract

**Background:**

Musculin (MSC) is a basic helix-loop-helix transcription factor that inhibits myogenesis during normal development and contributes to the differentiation defect in rhabdomyosarcoma. As one of many transcription factors that impede myogenesis, its binding on a genome-wide scale relative to the widespread binding of the myogenic factor MyoD is unknown.

**Methods:**

Chromatin immunoprecipitation coupled to high-throughput sequencing was performed for endogenous MSC in rhabdomyosarcoma cells and its binding was compared to that of MyoD in the same type of cells.

**Results:**

MSC binds throughout the genome, in a pattern very similar to MyoD. Its binding overlaps strongly with regions enriched for acetylated histone H4, as well as regions that score high for DNase hypersensitivity in human myoblasts. In contrast to MyoD, MSC has a more relaxed binding sequence preference in the nucleotides that flank the core E-box motif.

**Conclusions:**

The myogenic inhibitor MSC binds throughout the genome of rhabdomyosarcoma cells, in a pattern highly similar to that of MyoD, suggesting a broad role in buffering the activity of MyoD in development and rhabdomyosarcomas.

## Background

The advent of high-throughput sequencing coupled to chromatin immunoprecipitation (ChIP-seq) has permitted the global assessment of DNA binding of numerous transcription factors. While some factors show a relatively restricted binding pattern near their regulated genes, others bind widely throughout the genome [1]. The basic helix-loop-helix (bHLH) gene MyoD, a key regulator for the specification and differentiation of skeletal muscle [2], shows widespread binding at tens of thousands of genomic locations [3]. In addition to directly regulating the transcription of genes associated with a subset of the locations it binds, MyoD binding also results in histone acetylation at its binding sites throughout the genome, demonstrating a biological consequence of its genome-wide binding [3].

The myogenic activity of MyoD can be inhibited by a variety of transcription factors, including other members of the bHLH protein family [4]. Inhibitory mechanisms take a variety of forms, including competition for protein partners [5,6], the occlusion of MyoD binding sites and transcriptional repression after DNA binding [7,8], and binding to MyoD itself [9]. Musculin (MSC) is a small bHLH inhibitor that functions with a variety of mechanisms. Like MyoD, MSC forms heterodimers with E-proteins. The MSC:E-protein heterodimer binds to E-boxes and inhibits myogenic reporters and MyoD-mediated myogenesis [10]. The activity of MSC is quite complex, however, with a critical role in the specification and survival of cells destined to become a subset of craniofacial muscles in mice [11], possibly through regulation of the expression of members of the myogenic regulatory factor (MRF) family such as *MyoD* and *Myf5*[12]. A similar, crucial role in craniofacial muscle development has been seen in zebrafish models [13], and the *Drosophila* ortholog of *musculin* is required for the specification of certain gut muscle cells [14]. There is also evidence that musculin is not restricted to expression in skeletal muscle and functions to affect the differentiation of non-myogenic cells [15-17]. Together these studies indicate that *musculin* might have either positive or negative activities in gene transcription depending on a variety of factors and cellular context.

Recently, we have shown that MSC competes with MyoD for the available pool of E-proteins in rhabdomyosarcoma cells [18], and that it occludes MyoD binding sites, interfering with myogenic activation [19]. Rhabdomyosarcoma (RMS) is a pediatric tumor of skeletal muscle that fails to undergo terminal myogenic differentiation properly. These tumors express MyoD [20] and many also express MSC [18]. Since the tumors appear to represent an arrested state of development of normal muscle cells undergoing the transition from proliferative myoblasts to terminally differentiated myotubes [18,19], this makes RMS cells an ideal system for comparing the binding of MSC and MyoD and further elucidating the ability of MSC to function as an inhibitor of differentiation.

We have previously performed ChIP-seq for MyoD in a cell culture model of embryonal RMS, RD cells [21], and we now report a genome-wide assessment of MSC binding in RD cells. Strikingly, MSC binds widely throughout the genome, in an overlapping but non-identical pattern to MyoD, reflecting an overlapping but not identical E-box sequence specificity. The substantial direct overlap of MSC and MyoD sites together with the close proximity of many MSC- and MyoD-specific sites suggests that MSC has the potential for broadly modulating MyoD activity in normal development and in rhabdomyosarcomas.

## Methods

### Cell culture and construct preparation

RD cells were obtained from the American Type Culture Collection (ATCC), and all analyses were performed on cells that originated from low passage number frozen aliquots. RD cells were maintained in DMEM with 10% bovine calf serum and 1% Pen-Strep (Gibco). MSC with a tandem affinity purification (TAP) tag was constructed by cloning the coding sequence for MSC in-frame with a TAP-tagged pBabe plasmid so that the TAP tag is N-terminal to MSC.

### Chromatin immunoprecipitation and ChIP-seq

Chromatin immunoprecipitation (ChIP) was performed in RD cells with an approach that has been described previously [3]. Antibodies used were as follows: MyoD [22], MSC (Santa Cruz, sc-9556X). Quantitative PCR (qPCR) was performed using SybrGreen from Bio-Rad on an Applied Biosystems 7900HT. Enrichment was calculated as the percentage of input in samples with antibody divided by the percentage of input in matched samples without antibody. Primer sequences for site-specific confirmatory ChIP were as follows: A – f: gcttgatgatgcttgcagaa r: cggagaggatcatgtaactgc; B – f: ctggtccctttcaggagaca r: gccgtccatctaaaggtcaa; C – f: aatgacaagcactcgcacaa r: atcgagaagttgcgtgcttt; D – f: atctggaatgccttctgtgg r: attgcctaggaagggacaca; E – f: gcgacgagctccacatctac r: aggatgcccatgactttgag; F – f: ctcaccatccgaccaagagt r: ggggtcacgtgtgtatgaga.

### Liquid chromatography and mass spectrometry

The isolation of complexes associated with TAP-tagged MSC was performed identically to prior experiments [18], but MSC-associated complexes were only purified singly through tobacco etch virus (TEV)-mediated elution. Peptides were digested with trypsin before loading on a ThermoFinnigan LTQ FT and undergoing liquid chromatography coupled to tandem mass spectrometry (LC-MS/MS). The data were searched using X!Comet.

### Electrophoretic mobility shift assays

Shift assays were performed as described previously [23]. Proteins were transcribed and translated in vitro from CS2-based plasmids using a rabbit reticulocyte lysate kit (Promega). Probe sequences were as follows (forward sequences only listed, reverse complement sequences not shown): MSC-specific: cggccgaccagctggagatcct; -1 position mutation (mut): cggccgagcagctggagatcct; -1/+1 position mut: cggccgagcagctgcagatcct; MSC-specific T mut: cggccgtccagctggagatcct; -1/+1 T mut: cggccgtgcagctgcagatcct; CG E-box: cggccgaccacgtggagatcct; B1: gatccccccaacacctgctgcctga.

### Peak calling

Sequences were extracted by GApipeline-0.3.0. Reads mapping to the X and Y-chromosomes were excluded from our analysis. Reads were aligned using BWA to the human genome (hg19). Duplicate sequences were discarded to minimize the effects of PCR amplification. Each read was extended in the sequencing orientation to a total of 200 bases to infer the coverage at each genomic position. Peak calling was performed by an in-house developed R package, which models background reads by a negative binomial distribution as previously described [24]. Peaks in the MyoD and MSC samples that overlapped with peaks in the RD no antibody cell-type specific control sample at a *P* value cutoff of 10^-5^ were removed from the analysis.

### Motif analysis

We applied an in-house developed Bioconductor package motifRG for discriminative *de novo* motif discovery as previously described [3,25]. To find discriminative motifs for MSC-specific peaks, we selected MSC-specific and MSC- and MyoD-shared peaks. Specific peaks were defined as peaks present for one transcription factor with a *P* value cutoff of 10^-10^ and absent for the other with a *P* value cutoff of 10^-4^. Shared peaks were present for both factors with a *P* value cutoff of 10^-10^.

### *P* value peak overlap analysis

We adopted a nonparametric rank-based paradigm to compare two ChIP-seq samples as previously described [24]. We ranked all peaks by their *P* values and grouped ranks into bins of 3,000 (that is, the top 3,000 peaks, then the top 6,000 peaks, and so on). Then we computed the fraction of top *x* peaks in a sample that overlap with the top *y* peaks in another sample, where *x* and *y* vary from 3,000 to 30,000, and *y* is equal to or greater than *x*.

## Results

### Musculin and MyoD have overlapping, but non-identical, genome-wide binding patterns

To compare the binding pattern of the bHLH myogenic inhibitor MSC to that of the myogenic activator MyoD, ChIP-seq for endogenous MSC was performed in RD cells under growth conditions. MSC binds at a comparable number of sites as MyoD and with a similar genomic distribution, although there was a slightly greater enrichment of MSC binding in the region surrounding the transcription start site (TSS) compared to MyoD (Table 1), possibly reflecting the GC-rich nature of promoters and the preferred MSC E-box (see below). As with MyoD, MSC was found to bind widely at regions outside of those generally thought of as gene related, binding to a high degree (approximately 40% of all sites) in intergenic regions. A number of sites identified as being specifically and strongly enriched for either MyoD or MSC by ChIP-seq were tested with biologically independent site-specific ChIP, and factor-specific enrichment in agreement with the ChIP-seq data found at all sites (Additional file 1: Figure S1).

**Table 1:** Number and genomic location of musculin and MyoD ChIP-seq peaks in RD cells

**Factor**	**Number of peaks**			**Genomic location (fraction of peaks)**^**a**^							
	*P* value cutoff^b^			Promoter^c^	Proximal promoter^d^	3 Prime^e^	Exon	Intron	Upstream^f^	Downstream^g^	Intergenic^h^
	10^-5^	10^-7^	10^-10^								
Musculin	54901	39036	25688	0.165	0.231	0.029	0.204	0.563	0.209	0.170	0.423
MyoD [21]	50320	35203	24501	0.110	0.175	0.027	0.154	0.560	0.187	0.164	0.405

^a^The fraction of MyoD and musculin peaks found in each listed type of genomic region are given. Note that categories are not mutually exclusive, and a single peak may be included in multiple categories.

^b^Three *P* value cutoffs were used to evaluate whether ChIP-seq reads are considered a ‘peak’ and included in the count of the total number of peaks.

^c^+/−500 bp from the transcription start site (TSS).

^d^+/−2 kb from the TSS.

^e^+/−500 nucleotides from the end of the transcript.

^f^–2 kb to −10 kb upstream of the TSS.

^g^+2 kb to +10 kb from the end of the transcript.

^h^>10 kb from any annotated gene.

MSC heterodimerizes with E-proteins to bind to E-boxes [10], and we have previously shown by LC-MS/MS that the E-protein E12 associates with MSC in RD cells, while MyoD does not associate with MSC [18]. To further confirm that the ChIP-seq data represent distinct MyoD or MSC bHLH dimers, a TAP-tagged MSC was created. This was shown to maintain biological activity as measured by its ability to repress myogenic reporters and bind E-boxes in electrophoretic mobility shift assays (EMSAs) (data not shown). The tagged MSC was then introduced stably into RD cells through retroviral transduction and MSC-associated complexes pulled down and subjected to LC-MS/MS. As expected, all E-proteins were found to associate with MSC, while there was no indication of a MSC: MyoD interaction (Additional file 2: Table S1).

**Figure 1:**
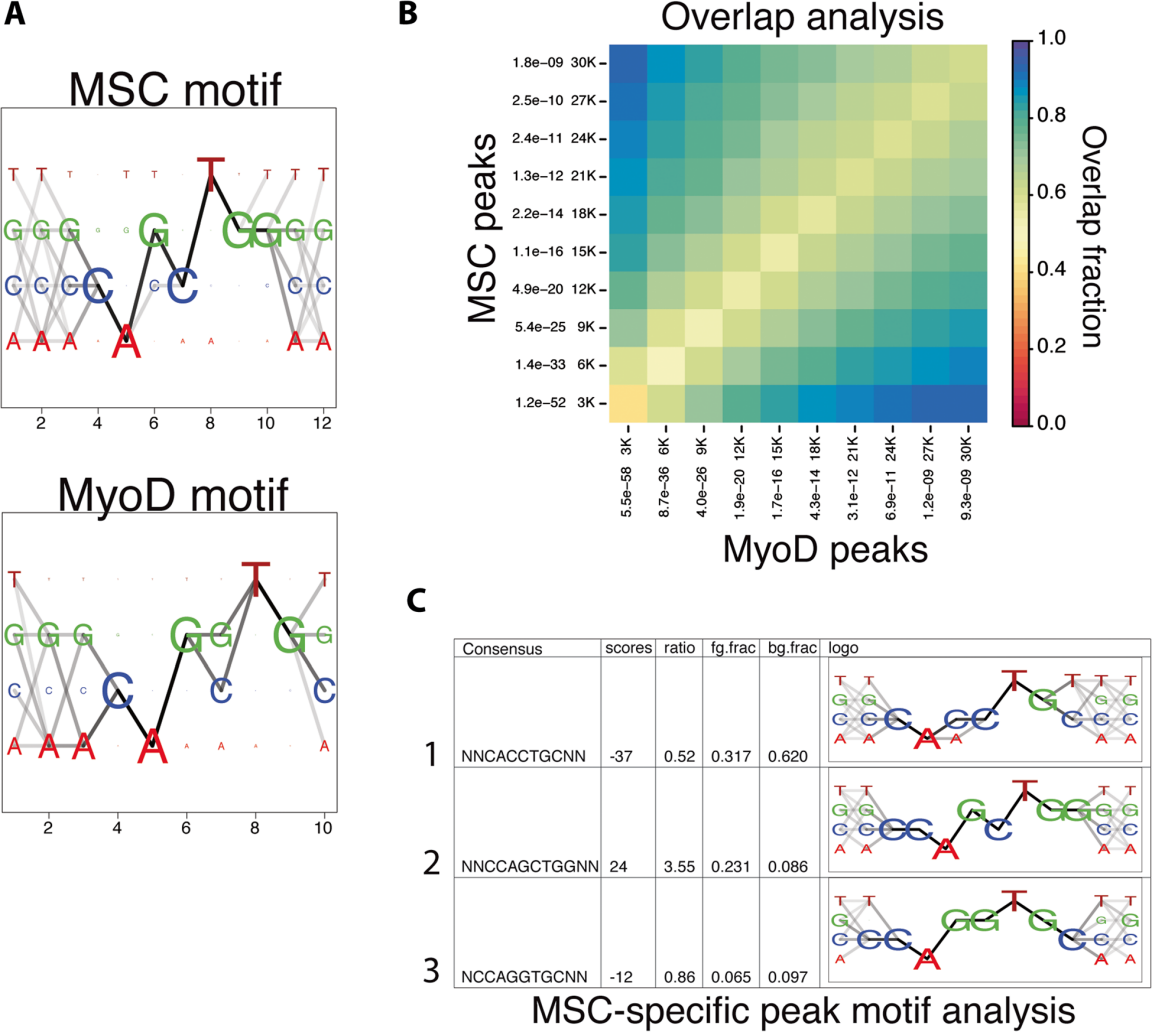
**MSC has similar, but non-identical, DNA binding characteristics to MyoD and binds at many of the same genomic locations.** (**A)** E-box motif enrichment of MSC and MyoD bound sites in RDs identifies a similar preference for central dinucleotide identity (GC and GG), but differing preferences in the E-box flanking nucleotides. **(B)** Comparison of the top 30,000 MyoD and MSC peaks in RDs demonstrates substantial overlap in the sites bound by each factor. Peaks were ranked by *P* value, and grouped into bins that increase by 3,000 peaks each time (that is, first the 3,000 most significant peaks are considered, than the 6,000 most significant, and so on). The fraction of the overlap is indicated by color, as depicted in the legend. **(C)***De novo* motif analysis of peaks specific to MSC identifies an 8 bp motif (row 2) enriched at MSC-specific binding sites. The motif analysis compared MSC-specific binding sites to those sites that bound both MyoD and MSC. bp, base pair; fg.frac, bg.frac: fraction of foreground/background sequences that contain at least one motif occurrence; MSC, musculin; ratio, enriched/depleted ratio of motifs.

A motif analysis of the binding site preferred by MSC found strong enrichment for binding at a GC core E-box (Figure 1A, top), one of the two E-box cores we previously identified as being preferred by MyoD (Figure 1A, bottom). In contrast to MyoD, MSC exhibits a strong nucleotide preference for a ‘G’ at the first nucleotide after the E-box (CAGCTGG), designated position +1 relative to the E-box. Also notable was a difference in the sequences enriched at the two positions immediately before the E-box, designated positions −1 and −2 relative to the E-box. We have previously shown that MyoD:E and NeuroD2:E heterodimers show a flanking preference for G or A in the −1 and −2 positions [3,24], whereas the MSC motif does not demonstrate a similarly strong preference at these positions (Figure 1A, positions 2 and 3).

**Figure 2:**
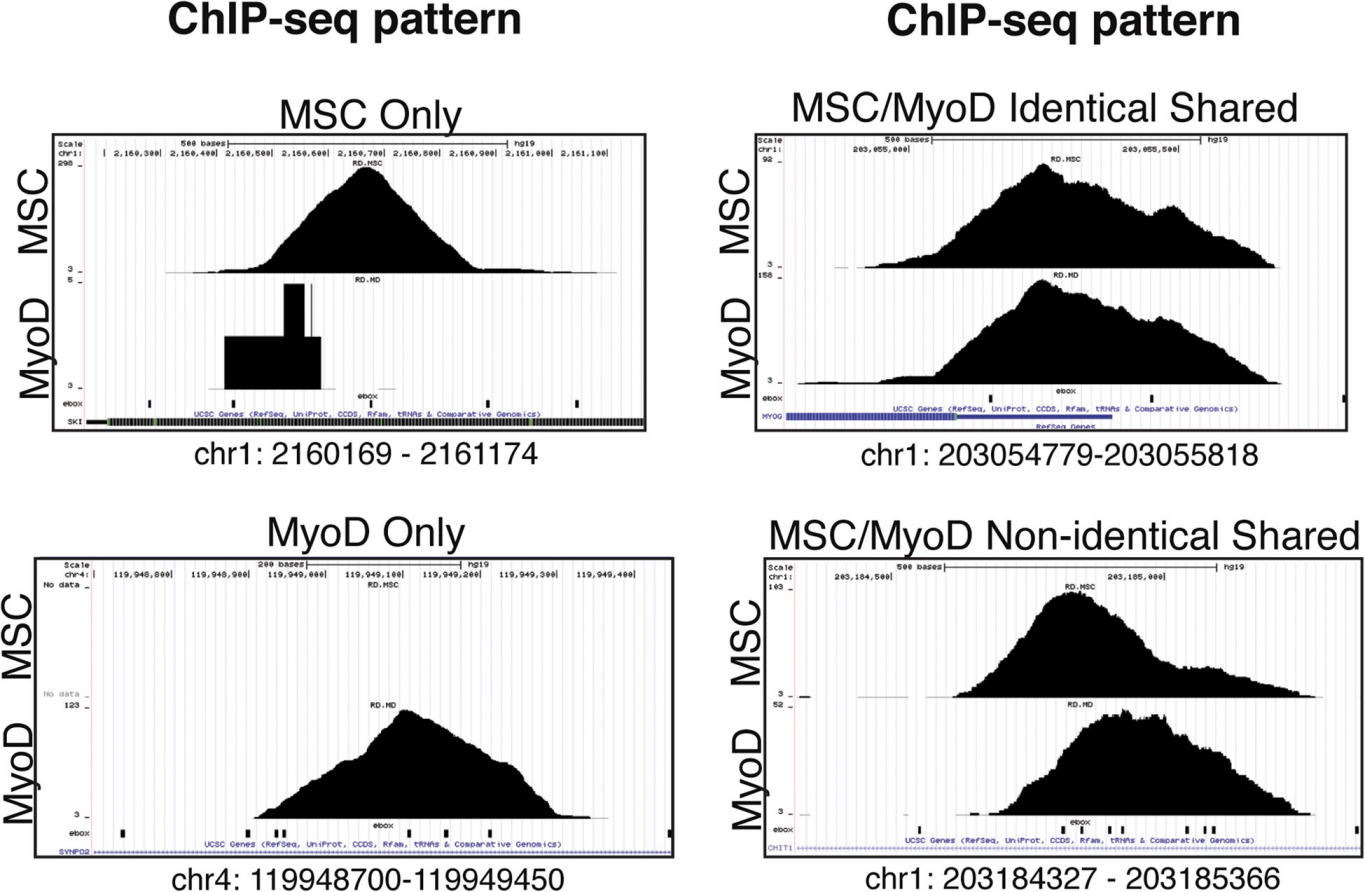
**MyoD and MSC bind at unique identical and overlapping but non-identical sites in the genome.** MSC and MyoD have both unique and overlapping binding patterns at various sites in the genome. Screenshots are shown from the UCSC Genome browser for MyoD and MSC ChIP-seq results at four distinct genomic locations (indicated below each panel, representing positions in hg19). The identity of the bHLH factor is indicated along the left, and E-boxes are represented as black marks along the bottom of each panel. Note that the number of MyoD reads in the ‘MSC only’ panel is five, in contrast to 298 reads for MSC, and are not centered on an E-box, and thus do not likely represent true MyoD binding. bHLH, basic helix-loop-helix; ChIP-seq, chromatin immunoprecipitation coupled to high-throughput sequencing; MSC, musculin.

As anticipated from the motif analysis, MyoD and MSC showed overlapping but not identical binding locations in the genome. MyoD and MSC peaks were assigned to sequential cumulative bins of 3,000 peaks based on rank by *P* value and the percentage overlap ranged from approximately 40% to 80% (Figure 1B). A motif analysis of sites that were found to bind only MSC (MSC-specific) in comparison to sites bound either solely by MyoD (MyoD-specific) or by both MyoD and MSC (shared) identified a strong enrichment for C at the −1 position and G at the +1 position, giving an 8-bp motif of CCAGCTGG (Figure 1C). Examination of the ChIP-seq data at specific loci identified sites bound only by one of the factors, sites bound by both factors in an apparently identical pattern, and sites bound by each factor in closely overlapping but non-identical binding patterns (Figure 2). The closely overlapping but distinct patterns suggests each factor is binding to a distinct E-box in the region; however, this is identified as an ‘overlap’ in the analysis shown in Figure 1B.

### Musculin binding is enriched at DNase hypersensitive genomic regions and regions with higher levels of histone acetylation

**Figure 3:**
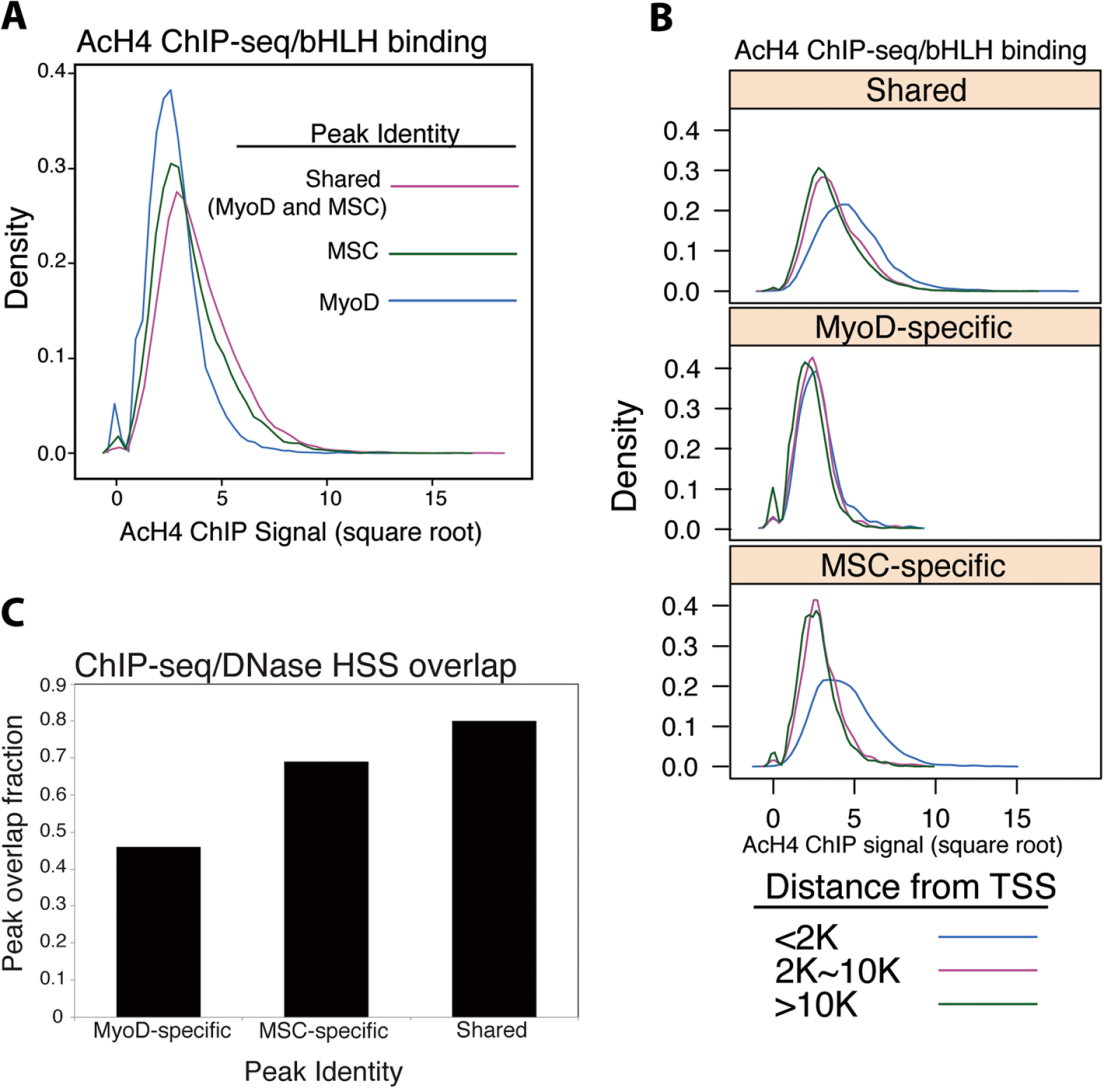
**MSC binding is associated with open chromatin.** (**A)** Sites bound by MyoD and MSC are associated with acetylated histones. ChIP-seq for acetylated histone H4 (AcH4) was performed in RD cells and density plots constructed to compare the square root of the AcH4 value at all sites bound by MSC, MyoD or both factors. **(B)** MSC-specific and MyoD/MSC shared peaks are associated with higher levels of AcH4 near the transcription start site (TSS) of genes compared to MyoD-specific peaks. Density plots were constructed as in (A) for categories of peaks first split by peak identity (MyoD, MSC, shared), then subcategorized on distance from the nearest TSS. **(C)** Sites bound by MSC in RD cells overlap with DNase hypersensitive (HSS) sites in normal human myoblasts. Publicly available DNase HSS data from human myotubes were compared to the sites bound by MyoD and MSC in RD cells. Data for each factor category (for example, MSC-specific) are plotted as the fraction of peaks that overlap with locations that have a signal in the HSS data (that is, the graphed fraction = 1 – fraction of peaks at HSS score of ‘0’). AcH4, acetylated histone H4; bHLH, basic helix-loop-helix; ChIP, chromatin immunoprecipitation; ChIP-seq, chromatin immunoprecipitation coupled to high-throughput sequencing; HSS, hypersensitive; K, thousands of bp; MSC, musculin; TSS, transcription start site.

We have previously shown that MyoD binding induces histone acetylation at binding sites throughout the genome [3]. To test the hypothesis that the genome-wide binding of MSC might inhibit acetylation in either a global manner or at some subset of MyoD-bound locations, we performed ChIP-seq for acetylated histone H4 (AcH4) from RD cells under conditions similar to the MyoD and MSC ChIP-seq data. AcH4 enrichment was examined at peaks identified as MSC-specific, MyoD-specific and shared. Surprisingly, the highest levels of AcH4 enrichment showed a stronger association with MSC peaks, both MSC specific and shared (Figure 3A). This trend became even more evident when peaks were grouped based on distance from the nearest gene TSS. While MyoD-specific peaks showed essentially identical AcH4 enrichment regardless of their location relative to a TSS, MSC-specific and shared peaks showed a strong shift to higher AcH4 enrichment at peaks located closer to a TSS (<2 kb from the nearest TSS) (*P* value of the difference between MSC-specific and MyoD-specific peaks: 1 × 10^-45^, *P* value for MSC-specific versus shared peaks: 1 × 10^-7^) (Figure 3B). MSC binding did not correlate, either positively or negatively, with genes that we have previously identified as being differentially regulated in RD cells compared to normal myogenic cells [21] (data not shown).

Given the lack of a global effect on gene expression, we hypothesized that the association with AcH4 might simply reflect binding of MSC at regions of open chromatin. The MyoD-specific, MSC-specific and shared peaks in the RD cells were compared to publicly available DNase hypersensitivity data from human myoblasts. Shared peaks had the highest proportion of peaks that overlapped with DNase hypersensitive sites (shared: approximately 80%, MSC-specific: approximately 70%, MyoD-specific: approximately 50%) (Figure 3C), and this relation held across the entire range of hypersensitive values (Additional file 3: Figure S2A).

MyoD-specific peaks seemed to have a surprisingly low level of association with hypersensitive sites, but subcategorizing the MyoD-specific peaks based on whether they were unique to RD cells, or common to RDs and human myotubes [21] revealed that common peaks were generally associated with hypersensitive sites, and peaks unique to RDs were not (Additional file 3: Figure S2B). We have previously shown that differences in MyoD binding between myotubes and RD cells can be correlated with differences in E-box accessibility between the cell types [21]. This suggests that the MyoD peaks specific to RMS, that is, not present in primary skeletal muscle cells, represent binding by MyoD to E-boxes that are normally inaccessible to bHLH binding in primary muscle cells and were therefore not identified as lying in HSS regions in the primary muscle cell dataset. Taken as a whole, the above data identify MSC binding as largely occurring in the context of areas of open and accessible chromatin.

### Musculin dimers have less restrictive binding site preferences at flanking nucleotides than MyoD dimers

**Figure 4:**
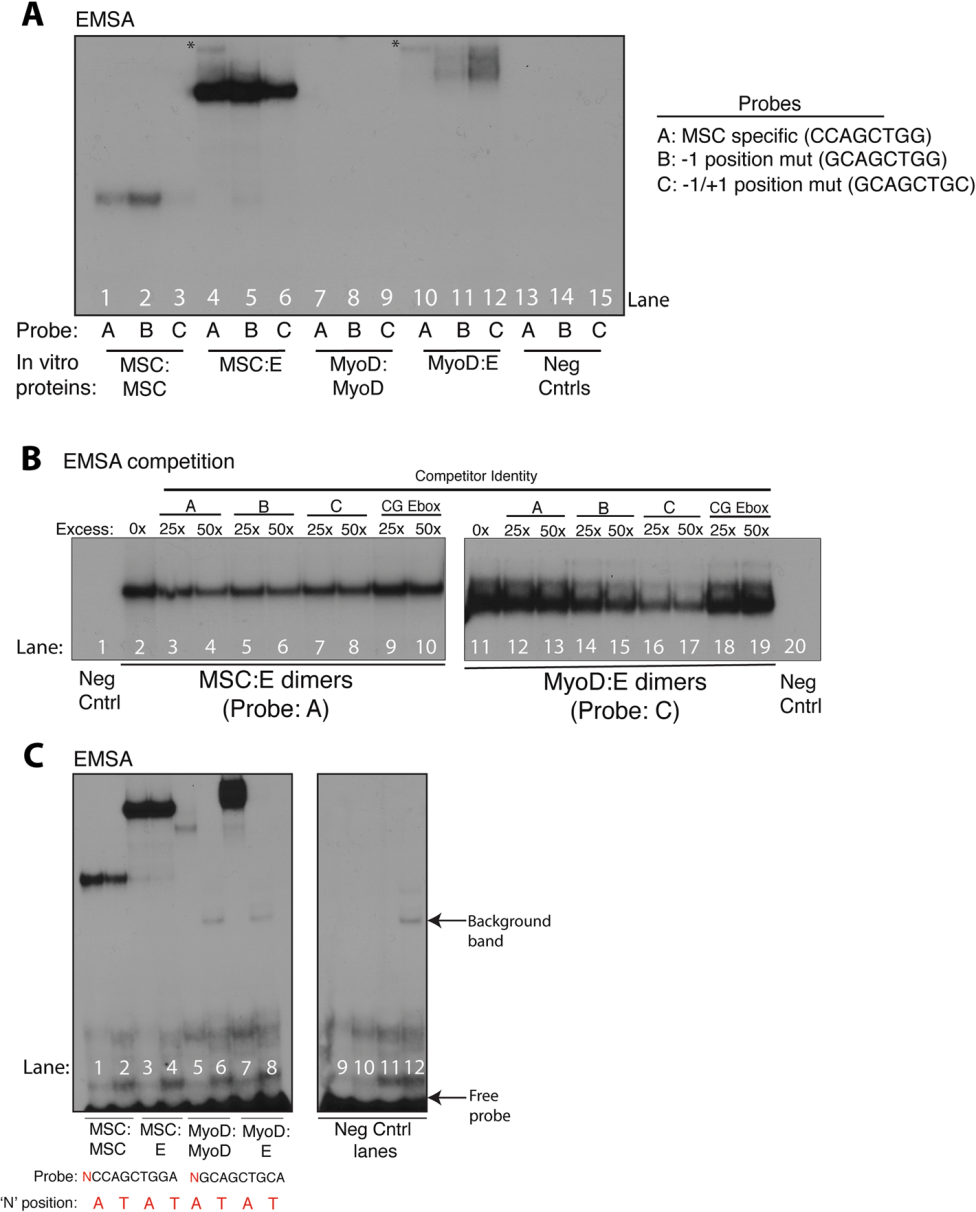
**MSC dimers have relaxed requirements for flanking sequence compared to MyoD dimers.** (**A)** MyoD homodimers and MyoD:E-protein heterodimers do not bind well to MSC-specific sequences, but bind after a small number of sequence changes. Electrophoretic mobility shift assays (EMSAs) were performed using in vitro translated proteins and probes as indicated. The asterisks indicate the location of what are, judging by their relative mobility, small amounts of E-protein homodimers. **(B)** MSC heterodimers can be competed off a preferred binding site equally well by competitors with variations in their flanking sequence, while MyoD heterodimers cannot. MyoD:E and MSC:E heterodimers were subjected to competition by excesses of cold probes as indicated. 25× and 50× refer to the excess mass of cold probe relative to hot probe. Variations in competitor sequences are indicated, and ‘CG Ebox’ refers to a probe with an inverted central dinucleotide sequence that abolishes all binding of MyoD and MSC. **(C)** Single nucleotide changes in flanking sequence can completely abrogate MyoD dimer binding, but still be permissive of MSC dimer binding. Shift assays were performed using proteins and probes as indicated. Each type of dimer combination was run in two lanes, with one lane having a probe with ‘A’ in the −2 position relative to the E-box, and the other lane having a probe with ‘T’ in that position, as indicated in red. All shifts were performed using a sufficient excess of probe so that visible free probe was present for all lanes (not shown in 4A and 4B). All probe counts were quantitated before addition to ensure there were roughly equivalent amounts in all compared lanes. Negative control lanes indicate lanes where probes were tested with an in vitro translated empty CS2 vector to identify any non-specific binding. EMSA, electrophoretic mobility shift assay; MSC, musculin.

Electrophoretic mobility shift assays with in vitro translated proteins were performed to further investigate the sequence preference of MyoD and MSC dimers using the sequence from a MSC-specific peak at the SKI gene that contained the MSC-specific consensus 8-bp motif (CCAGCTGG). Shifts comparing binding of MyoD:E and MSC:E heterodimers demonstrated that MSC heterodimers could bind to the 8-bp motif or a probe in which the −1 position was changed from a ‘C’ to a ‘G’ (−1 mut), making it more reflective of the core MSC motif from Figure 1A (Figure 4A, compare lane 4 to 5). Binding of MSC to a probe in which both −1 (‘C’ to ‘G’) and +1 (‘G’ to ‘C’) had been changed (−1/+1 mut) still occurred, but at reduced levels (Figure 4A, compare lanes 4 and 5 to 6). In contrast, the ability of MyoD:E heterodimers to form complexes improved as the probe was shifted away from the 8-bp MSC motif (Figure 4A, compare lane 10 to 12). Taken together with the motif analysis identifying the differences at positions −1 and −2, this suggests that the sequence preference for dimer binding is more stringent for dimers containing MyoD than those containing MSC, even with a common dimer partner. Similar results were observed with homodimers of both MSC and MyoD, though both types of homodimers formed more weakly compared to their heterodimer counterparts (Figure 4A, compare lane 1 to 3 and 7 to 9, data not shown).

To further test this hypothesis, competition assays were performed on MSC and MyoD heterodimers. As expected, MSC was competed off the MSC-specific 8-bp motif equally well by cold competitors with the consensus 8-bp motif, –1 mut, or −1/+1 mut (Figure 4B, left panel, compare lanes 3 and 4 to 5 and 6, and to 7 and 8), suggesting relatively similar affinity. MSC was not competed with a sequence in which the core nucleotides of the E-box were inverted to a ‘CG’ from ‘GC’ (Figure 4B, left panel, compare lanes 9 and 10 to 2), demonstrating sequence specificity of the competition assays.

In contrast, MyoD heterodimers were only effectively competed by sequences at which it had formed visible complexes (Figure 4B, right panel, compare lanes 16 and 17 to 12 and 13, and to 14 and 15), and even a single nucleotide change had a notable impact on competition (compare −1/+1 mut to −1 mut). As with MSC, MyoD:E heterodimers failed to form on the CG core E-box (data not shown).

In addition to the relaxed preference at the positions immediately flanking the E-box, relative to MyoD, MSC also exhibited a sharp difference in response to sequence changes at the −2 position. The inclusion of a ‘T’ at the −2 position is permissive for MSC heterodimer binding (Figure 4C, compare lane 3 to 4), but absolutely abolishes binding of MyoD heterodimers (Figure 4C, compare lane 7 to 8), with similar results seen with the homodimers (Figure 4C, compare lane 1 to 2 and 5 to 6).

## Discussion

Our genome-wide comparison of the DNA binding characteristics of MyoD and MSC reveals that, even though MSC is one of multiple myogenic inhibitors and might be expected to bind at only a subset of all MyoD binding locations, it binds at a comparable number of sites as MyoD, with a similar, but non-identical binding site preference. Even though MSC heterodimerizes with the same E-proteins as MyoD and shares the same sequence preference at the central dinucleotide of E-boxes, it has less sequence preference for the positions that flank E-boxes than other bHLH dimers we have reported [3,24]. It should be noted that the electrophoretic mobility shift assays were performed using in vitro translated proteins, and thus would not reflect the effect of any post-translational modifications that may occur in vivo. However, the in vitro binding preferences reflect those preferences seen in the in vivo ChIP-seq results. Additionally, work with other bHLH factors has demonstrated excellent correlation between binding sites identified by ChIP-seq and binding seen with EMSA [24]. Overall, the broad overlap of MyoD and MSC binding indicates a potential for MSC to buffer the binding and activity of MyoD broadly, as well as other E-box binding factors.

We have previously shown that MSC can inhibit MyoD-mediated activation of myogenic targets by occluding specific E-boxes [19], in addition to competing for a limiting pool of E-proteins [18]. We have proposed that this activity controls the growth-versus-differentiation decision point in myogenic cells, serving as a component of interlocking oscillating regulatory circuits that keep myogenic cells balanced between proliferation and terminal differentiation [19].

This model suggests that the relationship between bHLH proteins and target sites is highly dynamic, in which dimers form and dissociate, from both their protein partners and DNA binding sites, resulting in a fluctuating expression of gene targets. In turn, some subset of such targets feed back on the process to regulate growth and differentiation appropriately. MSC has a very similar core binding motif as MyoD dimers but a greater degree of flexibility for flanking nucleotides, which could reflect a lower need for tight regulation of the specific E-boxes MSC can bind to compared to MyoD and be the mechanism by which MSC acts to broadly sequester E-proteins and occupy potential MyoD binding sites. Other factors have also been suggested as having an inhibitory function during myogenesis by binding at E-boxes [26,27], further potentially increasing the complex nature of the interplay occurring at bHLH binding sites. The similar core motif requirements for MyoD and MSC ensure that MSC binds at many sites regulated by MyoD, and the +1 ‘G’ preference of the MSC motif increases the likelihood of it targeting E-boxes located in GC-rich gene promoter regions. While the gene regulation analysis did not identify a global role in gene suppression, MSC does modulate MyoD activity at the myogenic microRNA miR-206 [19], and it may have a similar role at many other MyoD regulated genes, an effect that would not be discernible with our current analysis.

This model also offers possible insight into the ability of MSC to serve apparently as either a positive or negative regulator of transcription. With a component of MSC’s repressive activity appearing to be through interference with DNA binding by other bHLH factors, MSC activity could be substantially different depending on the individual cellular context, potentially interfering with the binding of inhibitory complexes. It is not known what histone modification enzymes MSC might recruit, nor is it clear how MSC activity would differ depending on the extent of competition by other bHLH proteins for binding partners and sites, and these additional parameters might contribute to a context-specific ability to serve as a positive or negative regulator.

Both the finding that MSC is associated with regions enriched for acetylated histone H4 in RD cells and DNase hypersensitive sites in normal myotubes suggests MSC generally binds at areas of open chromatin. It is unclear at this point why the notable enrichment is seen at sites closest to transcription start sites (<2 kb), though it is possible that part of this enrichment is due to the GC-rich nature of promoters and the binding site preference of MSC for an additional flanking ‘G’ compared to MyoD (Figure 1). While an effect by MSC on histone acetylation cannot be formally ruled out, mass spectrometry data did not identify any association with histone acetyltransferases (KLM, unpublished observations), suggesting that, in skeletal muscle cells, MSC is opportunistic in binding to areas of open chromatin, rather than instructing changes in chromatin structure. This would be consistent with the model proposed above, serving to assist MSC acting in a role as a dynamic competitor of MyoD function in the differentiation of skeletal muscle.

## Conclusions

The myogenic bHLH inhibitor musculin binds widely throughout the genome in RD rhabdomyosarcoma cells and has a broadly overlapping, but non-identical, set of binding sites and peaks as MyoD. Compared to the preferred MyoD E-box sequence, MSC has slightly less stringency for flanking sequence preference, permitting binding to a slightly broader set of E-boxes and potentially overlapping with other bHLH factors. Together with prior studies showing the ability of MSC to modulate MyoD activity at overlapping sites at specific promoters, these results suggest a broad potential for MSC to modulate the activity of MyoD, and perhaps other bHLH proteins, during normal development and in cancers.

## Abbreviations

AcH4: acetylated histone H4; bHLH: basic helix-loop-helix; bp: base pair; ChIP: chromatin immunoprecipitation; ChIP-seq: chromatin immunoprecipitation coupled to high-throughput sequencing; EMSA: electrophoretic mobility shift assay; HSS: hypersensitivity; kb: kilobase; LC-MS/MS: liquid chromatography coupled to tandem mass spectrometry; MRF: myogenic regulatory factor; MSC: musculin; mut: mutation; qPCR: quantitative PCR; RMS: rhabdomyosarcoma; TAP: tandem affinity purification; TEV: tobacco etch virus; TSS: transcription start site.

## Competing interests

The authors declare they have no potential competing interests.

## Authors’ contributions

KLM contributed to all experimental designs, performed all non-computational experiments and drafted the manuscript. ZY performed the ChIP-seq and all other computational analyses and contributed to both experimental design and data interpretation. APF performed the RD cell-type control ChIP-seq. SJT conceived the project, contributed to all experimental designs and edited the manuscript. All authors read and approved the final manuscript.

## Supplementary Material

Additional file 1: Figure S1 MyoD and MSC site-specific ChIP confirms the ChIP-seq results. Biologically independent site-specific ChIP was performed at three sites indicated by the ChIP-seq to be MyoD-specific binding sites, three sites indicated as MSC-specific, and one control location with no significant binding of either factor, as indicated by both the chart and the screenshots. The enrichment was calculated for each location as the percentage of input amplified in qPCR with antibody divided by the percentage of input amplified with no antibody, and the value is indicated at the top of each bar. Note that the *y*-axis is non-linear. Screenshots are from the UCSC genome browser, and the identity of the factor used in the ChIP, and the number of reads at the peak of occupancy are indicated along the side. ChIP, chromatin immunoprecipitation; ChIP-seq, chromatin immunoprecipitation coupled to high-throughput sequencing; MSC, musculin. Click here for file

Additional file 2: Table S1 LC-MS/MS identification of MSC-associated transcription factors in RD cells. Click here for file

Additional file 3: Figure S2 MSC binds sites associated with DNase hypersensitivity, and MyoD peaks found only in RD cells, not in normal myotubes, are associated with areas identified in myotubes as DNase-resistant. (A) Shared MyoD and MSC binding peaks are associated strongly with DNase hypersensitive (HSS) sites in human myoblasts. The overlap between ChIP-seq peaks and HSS data is graphed for the entirety of the range of HSS values. Values for a DNase signal of ‘0’ are equal to 1 – the fraction graphed in Figure 3C. The data are plotted as a cumulative distribution function, where a value on the *y*-axis represents the fraction of data that has a value equal to or less than the corresponding *x*-axis DNase HSS value. (B) MyoD-specific sites bound by MyoD only in RD cells, and not in human myotubes, overlap poorly with HSS sites in human myotubes. The MyoD-specific peaks from Figure 3C and (A) were further grouped into those peaks that were found both in RD cells and normal human myotubes (RD/myotube shared), and those found only in RD cells (RD-specific). As in Figure 3C, the data for each category (for example, RD-specific) are plotted as the fraction of peaks that overlap with sites that have some signal in the HSS data (that is, the graphed fraction = 1 – fraction of peaks at HSS score of ‘0’). ChIP-seq, chromatin immunoprecipitation coupled to high-throughput sequencing; HSS, hypersensitive; MSC, musculin. Click here for file
