# Supplementary figures and images for: Genome-wide binding of the basic helix-loop-helix myogenic inhibitor musculin has substantial overlap with MyoD: implications for buffering activity
Source: Skelet Muscle. 2013 Nov 1;3:26. doi: 10.1186/2044-5040-3-26 (PMC4177542; doi:10.1186/2044-5040-3-26)

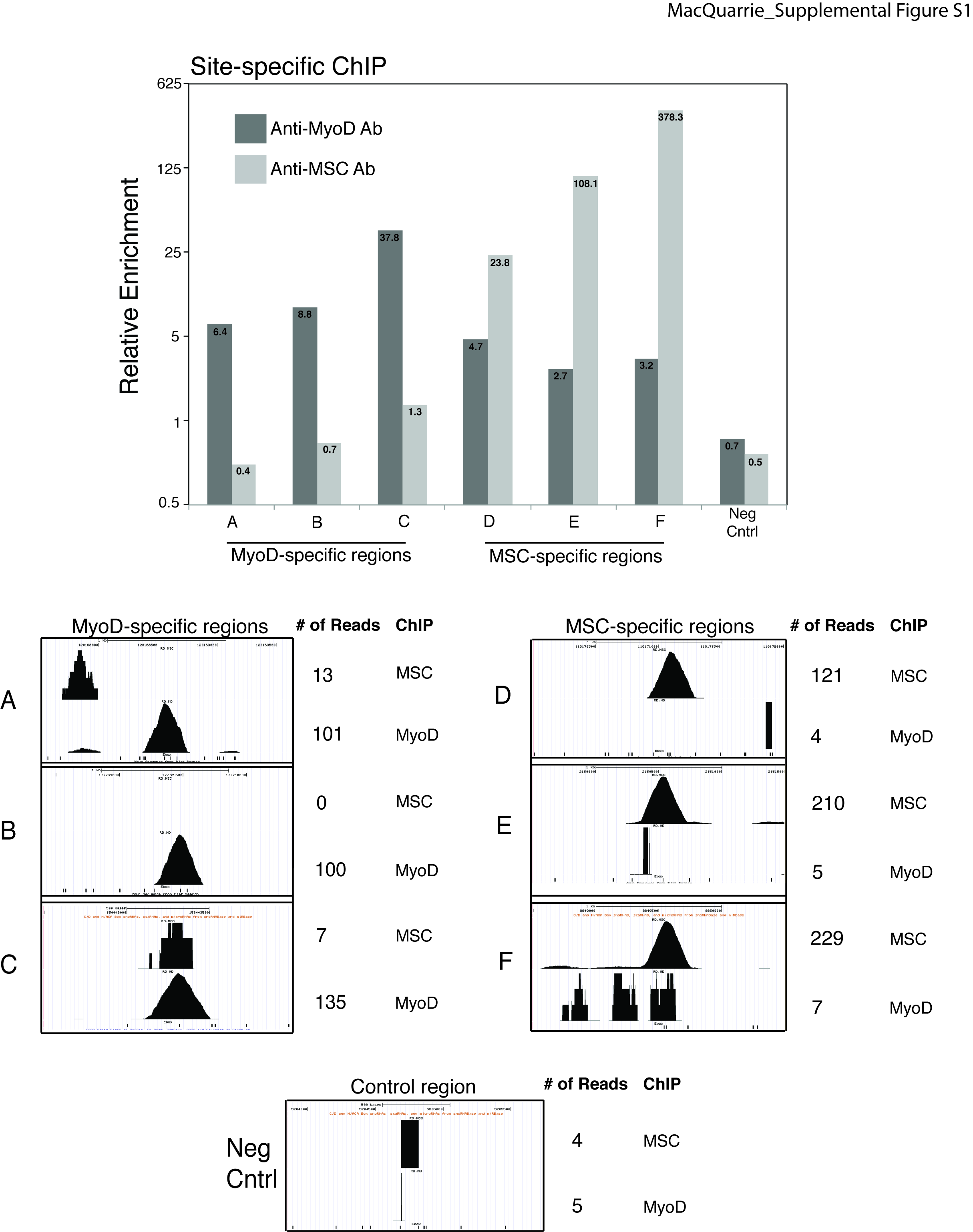

Supplement: Additional file 1: Figure S1 — MyoD and MSC site-specific ChIP confirms the ChIP-seq results. Biologically independent site-specific ChIP was performed at three sites indicated by the ChIP-seq to be MyoD-specific binding sites, three sites indicated as MSC-specific, and one control location with no significant binding of either factor, as indicated by both the chart and the screenshots. The enrichment was calculated for each location as the percentage of input amplified in qPCR with antibody divided by the percentage of input amplified with no antibody, and the value is indicated at the top of each bar. Note that the y-axis is non-linear. Screenshots are from the UCSC genome browser, and the identity of the factor used in the ChIP, and the number of reads at the peak of occupancy are indicated along the side. ChIP, chromatin immunoprecipitation; ChIP-seq, chromatin immunoprecipitation coupled to high-throughput sequencing; MSC, musculin. [file 2044-5040-3-26-S1.tiff]

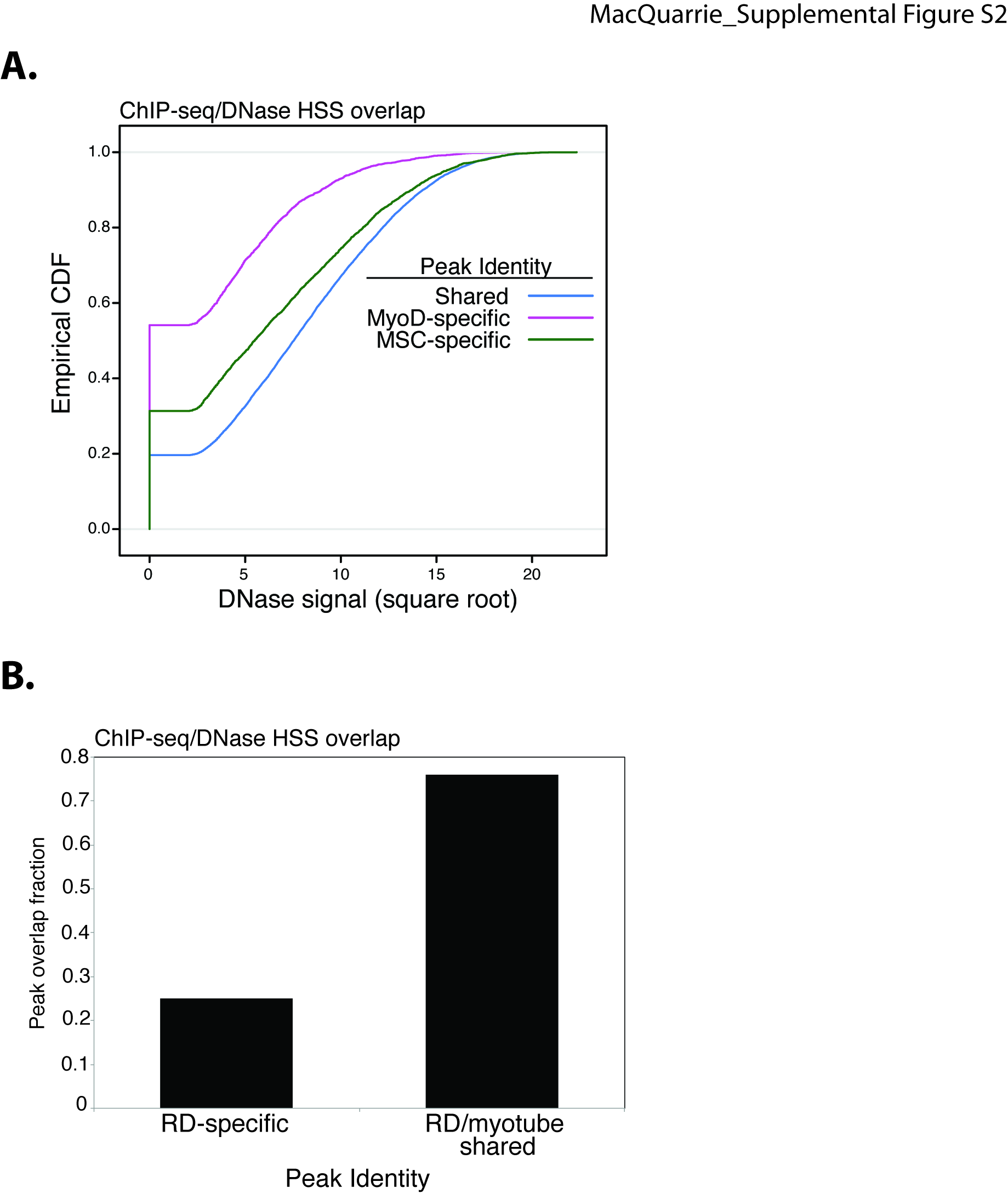

Supplement: Additional file 3: Figure S2 — MSC binds sites associated with DNase hypersensitivity, and MyoD peaks found only in RD cells, not in normal myotubes, are associated with areas identified in myotubes as DNase-resistant. (A) Shared MyoD and MSC binding peaks are associated strongly with DNase hypersensitive (HSS) sites in human myoblasts. The overlap between ChIP-seq peaks and HSS data is graphed for the entirety of the range of HSS values. Values for a DNase signal of ‘0’ are equal to 1 – the fraction graphed in Figure 3C. The data are plotted as a cumulative distribution function, where a value on the y-axis represents the fraction of data that has a value equal to or less than the corresponding x-axis DNase HSS value. (B) MyoD-specific sites bound by MyoD only in RD cells, and not in human myotubes, overlap poorly with HSS sites in human myotubes. The MyoD-specific peaks from Figure 3C and (A) were further grouped into those peaks that were found both in RD cells and normal human myotubes (RD/myotube shared), and those found only in RD cells (RD-specific). As in Figure 3C, the data for each category (for example, RD-specific) are plotted as the fraction of peaks that overlap with sites that have some signal in the HSS data (that is, the graphed fraction = 1 – fraction of peaks at HSS score of ‘0’). ChIP-seq, chromatin immunoprecipitation coupled to high-throughput sequencing; HSS, hypersensitive; MSC, musculin. [file 2044-5040-3-26-S3.tiff]
