# Supplementary material for: Genome-wide binding of the basic helix-loop-helix myogenic inhibitor musculin has substantial overlap with MyoD: implications for buffering activity
Source: Skelet Muscle. 2013 Nov 1;3:26. doi: 10.1186/2044-5040-3-26 (PMC4177542; doi:10.1186/2044-5040-3-26)
Supplement: Additional file 2: Table S1 — LC-MS/MS identification of MSC-associated transcription factors in RD cells. [file 2044-5040-3-26-S2.docx]

| **Gene Symbol** | **Number of Unique Peptides^a^** | **Percent of amino acids^b^** |
| --- | --- | --- |
| **HEB** | **11** | **29** |
| **E2A** | **21** | **40** |
| **E2-2** | **13** | **34** |
| MSC | 1 | 5 |
| EBF3 | 2 | 6 |
| LDB2 | 1 | 3 |
| PBX2 | 1 | 7 |

**Additional File 2. Supplemental Table S1. LC-MS/MS identification of MSC-associated transcription factors in RD cells.**

^a^Number of unique peptides lists the number of distinct peptides belonging to the indicated protein determined by the MS analysis to be present in the sample.

^b^Percent of amino acids indicates the amino acid percentage detected out of the total possible group of detectable amino acids for each indicated protein.

**Bold** entries indicate the members of the E-protein family.
